# Surgical site infection in spinal surgery: a bibliometric analysis

**DOI:** 10.1186/s13018-023-03813-6

**Published:** 2023-05-08

**Authors:** Xun Wang, Yanze Lin, Wenchao Yao, Aiqi Zhang, Liqing Gao, Fabo Feng

**Affiliations:** 1 Zhejiang Chinese Medical University, Hangzhou, 310053 Zhejiang China; 2 Department of Orthopaedics, The First People’s Hospital of Chun’an County, Hangzhou, 311700 Zhejiang China; 3 Center for Plastic and Reconstructive Surgery, Department of Orthopedics, Zhejiang Provincial People’s Hospital (Affiliated People’s Hospital, Hangzhou Medical College), Hangzhou, 310014 Zhejiang China

**Keywords:** Surgical site infection, SSI, Spinal infection, Citation analysis, Bibliometric, Web of Science

## Abstract

**Background:**

Surgical site infection (SSI) is a common complication in spinal surgery that can significantly affect the patient's prognosis. Despite advances in surgical techniques and infection control measures, SSI remains a considerable concern for healthcare providers and patients alike. In recent years, there has been a steady increase in studies related to SSI in spine surgery, leading to the publication of numerous informative articles. However, the current state and trends of research in the field of spinal SSI remain unclear. This study aims to conduct a bibliometric analysis of SSI-related articles in spine surgery to identify research status and trends. Meanwhile, we identify the top 100 most cited articles for further analysis.

**Methods:**

We searched for all articles related to spinal SSI in the Web of Science Core Collection, recording the publication year, country, journal, institution, keywords, and citation frequency for further analysis. In addition, we identified and analyzed the top 100 most cited articles.

**Results:**

A total of 307 articles related to spinal SSI were identified. All of these articles were published between 2008 and 2022, with the number of publications showing an increasing trend over the years. The related articles originated from 37 countries, with the USA contributing the most (*n* = 138). The institution with the highest number of publications and citations was Johns Hopkins University (14 articles; 835 citations). Among the journals, Spine had the highest number of articles (*n* = 47). The prevention of spinal SSI has been a research hotspot in recent years. Among the top 100 most cited articles, the most common research theme was the risk factors associated with spinal SSI.

**Conclusions:**

In recent years, research related to spinal SSI has attracted the attention of numerous clinicians and scholars. As the first bibliometric analysis of spinal SSI, our study aims to provide pragmatic guidance for clinicians to learn the research status and trends in this field and improve their vigilance toward SSI.

## Introduction

Surgical site infection (SSI) after spinal surgery is the third most common complication in spinal surgery, after pneumonia and urinary tract infections [1]. SSI can be divided into superficial, deep, and organ space SSI, depending on the site of infection. Superficial SSI refers to infections that involve only the skin or subcutaneous tissue around the incision; deep SSI refers to infections that penetrate the fascia or muscle layer; while organ space SSI refers to infections that involve the surgical site outside of the skin, fascia, and muscle layer [2]. The incidence of SSI after spinal surgery is reported to be approximately 0.7–16.1% [3, 4]. The most common pathogens causing SSI are *Staphylococcus aureus* and *Staphylococcus epidermidis* [5]. SSI can worsen the patient's condition, prolong hospital stay, seriously affect the patient's prognosis, and even lead to death, causing additional physical, mental, and economic burdens. There are multiple risk factors leading to SSI after spinal surgery. Previous studies have demonstrated that diabetes, previous SSI history, and obesity are significantly associated with the increased incidence of SSI [6]. The management of spinal SSI focuses on a multilevel comprehensive prevention strategy, including risk factors identification and perioperative prevention. For patients who have developed spinal SSI, surgical debridement and intravenous antibiotic therapy can be used during the acute phase of infection. However, in cases of chronic infection, these measures may be insufficient to eliminate the lesion, and removal of internal fixation and subsequent revision surgery may be necessary [7].

Up to now, numerous scholars have conducted comprehensive and in-depth research on the risk factors, etiology, diagnosis, prevention, and treatment of spinal SSI, and have published a large number of influential articles. However, the current research status, hotspots, and trends in spinal SSI remain undefined. Bibliometric is a qualitative and quantitative research method to explore the current status and trends in a particular field. To our knowledge, there is currently no bibliometric analysis focused on SSI after spine surgery. Therefore, the purpose of our study is to conduct a bibliometric analysis based on the published articles in spinal SSI to identify the research status and trends.

## Materials and methods

### Search strategy

All data involved in our study were obtained from the Web of Science Core Collection database. Identified the keywords and synonyms (surgical site infection; surgical site infections; surgical site infection; SSI), limited the article type to "Article" or "Review," with a language filter for English and a time range spanning from 1900 to 2022. The specific query was as follows: ((((TI = (surgical site infection OR surgical site infections OR surgical site infection OR SSI)) AND TI = (spine OR spinal OR lumbar OR thoracic OR cervical OR sacral)) AND DT = (Article OR Review)) AND LA = (English)) AND PY = (1900–2022).

The search was conducted on November 8, 2022. A total of 310 publications were retrieved, of which 307 were selected for further discussion after screening. We extracted and recorded the title, abstract, authors, publication year, country, journal, institution, keywords, and citation frequency for further analysis.

### Tools

The analysis of data was carried out using VOSviewer, Scimago Graphica, and Microsoft Excel 2016. VOSviewer and Scimago Graphica are capable of performing bibliometric data analysis and can provide complementary benefits when used together. We analyzed author, country, journal, institution, and keywords with the aforementioned software.

### Data extraction

Two authors independently extracted bibliometric indicators according to the specified query and discussed the differences until a consensus was reached. We used VOSviewer and Microsoft Excel 2016 to extract and analyze the data, including author, journal, institution, country, citations, keywords, and research trends. VOSviewer and Scimago Graphica were applied for data visualization.

## Result

### Publication trend

**Fig. 1:**
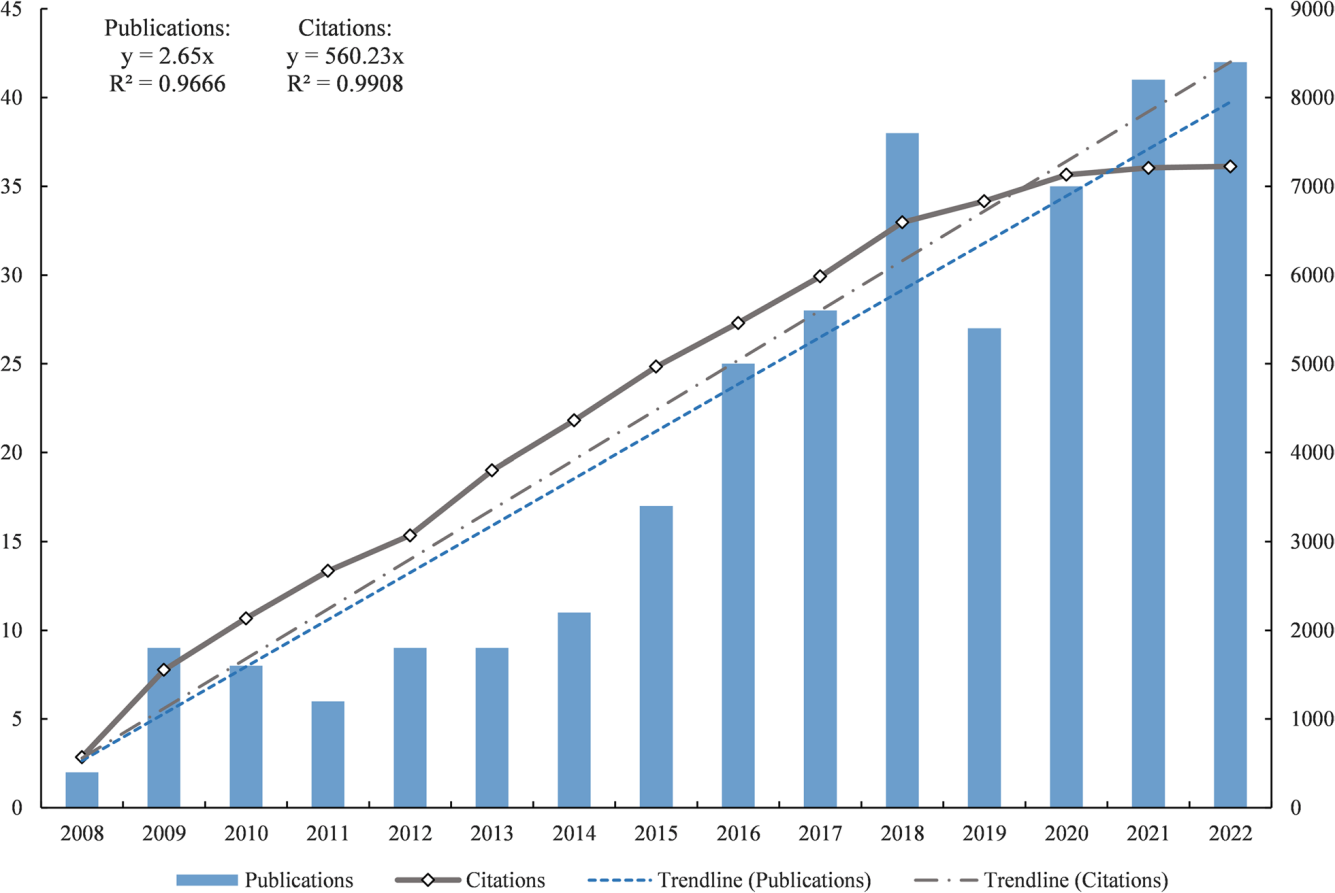
Annual publications and citations of spinal SSI research from 2008 to 2022

A total of 307 articles related to spinal SSI were identified. Overall, articles and citations have had a linear growth trend from 2008 to 2022 [*R*^2^(Publications) = 0.9666, *R*^2^(Citations) = 0.9908] (Fig. 1). Among all years, 2018, 2020, 2021, and 2022 were the most contributed years, with the annual publications has reached 35 or more. Judging from the frequency of citations, all publications were cited 7225 times, with an average of 23.53 citations per publication. All of these indicate that research on spinal SSI has been receiving increasing attention in recent years, as the number of related publications continue to rise.

### Country distribution

**Fig. 2:**
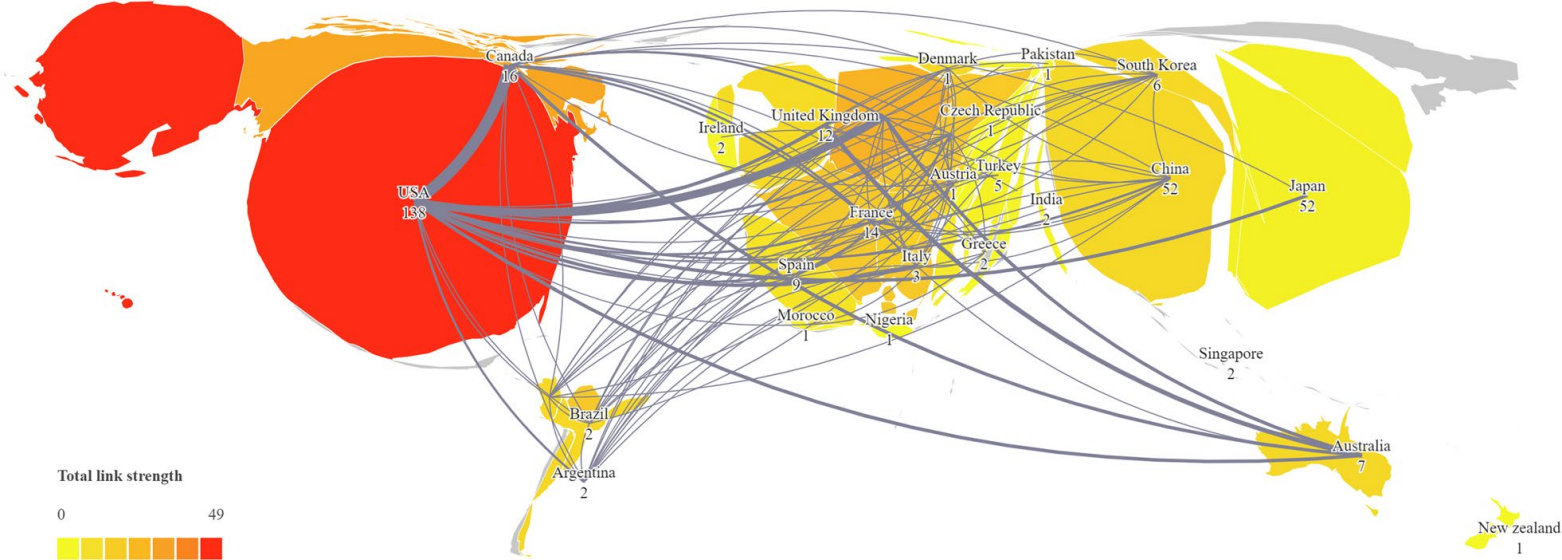
Visualization map of co-authorship among countries

All articles came from 37 different countries, of which the USA was the most contributed country with 138 articles, followed by Japan (*n* = 52), China (*n* = 52), and Canada (*n* = 16). Based on the advantage of the total number of articles, the USA was far ahead in total citation frequency with 4935 citations, compared to Japan (*n* = 791), China (*n* = 631), and Canada (*n* = 493). The co-authorship between countries was analyzed using VOSviewer and Scimago Graphica software. By drawing network visualization graphics, it was evident that most articles were published from North America and Asia. The USA stands as a prominent research hub for global spinal SSI, exhibiting a close-knit collaboration with Canada and the Netherlands, as indicated in Fig. 2.

### Institution distribution

A total of 505 institutions participated in the publication of SSI-related articles. The top 10 contributing institutions were Johns Hopkins University (US; *n* = 14), Mayo Clinic (US; *n* = 14), University of Tokyo (Japan; *n* = 11), Columbia University (US; *n* = 10), University of Washington (US; *n* = 9), Thomas Jefferson University (US; *n* = 8), University of British Columbia (Canada; *n* = 7), Japanese Red Cross Medical Center (Japan; *n* = 7), Musashino Red Cross Hospital (Japan; *n* = 7), and Saitama Medical University (Japan; *n* = 7) (Table 1). In terms of total citations, Johns Hopkins University ranked first (14 articles; 835 citations), followed by Washington University (five articles; 749 citations), Vanderbilt University (four articles; 526 citations), and Harvard University (five articles; 399 citations).

**Table 1:** The most contributed institutions on spinal SSI

Rank	Institutions	Publications	Total citations	Mean citations
1	Johns Hopkins University	14	835	59.64
2	Mayo Clinic	14	196	14.00
3	University of Tokyo	11	152	13.82
4	Columbia University	10	364	36.40
5	University of Washington	9	284	31.56
6	Thomas Jefferson University	8	339	42.38
7	University of British Columbia	7	160	22.86
8	Japanese Red Cross Medical Center	7	93	13.29
9	Musashino Red Cross Hospital	7	93	13.29
10	Saitama Medical University	7	93	13.29
11	University of California San Francisco	6	318	53.00
12	Childrens Hospital of Philadelphia	6	151	25.17
13	Sanraku Hospital	6	91	15.17
14	Tokyo Metropolitan Cancer & Infectious Diseases Center Komagome Hospital	6	91	15.17
15	Yokohama Rosai Hospital	6	91	15.17
16	Saitama Red Cross Hospital	6	63	10.50
17	Washington University (WUSTL)	5	749	149.80
18	Harvard University	5	399	79.80
19	Hebei Medical University	5	134	26.80
20	University of Pittsburgh	5	110	22.00

**Fig. 3:**
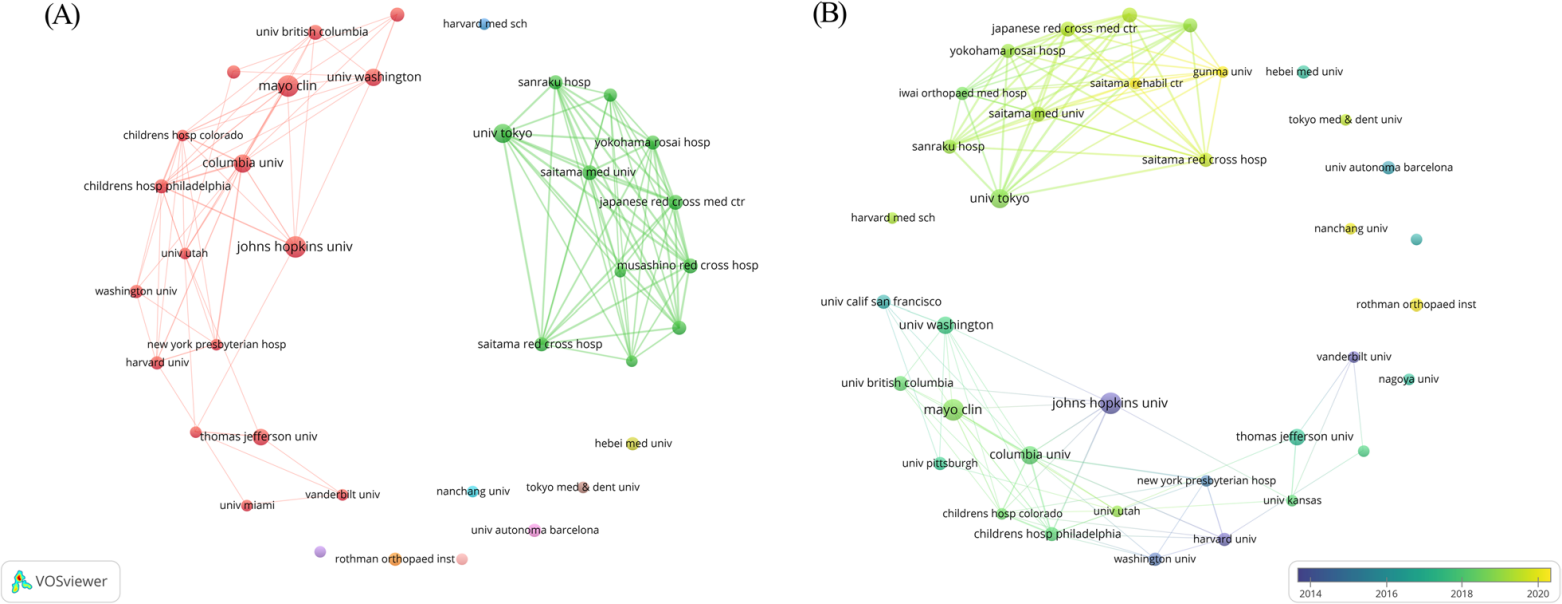
**A** Cooperation network among institutions on spinal SSI **B** Major research institutions over time from 2008 to 2022

We employed VOSviewer to construct network visualization and overlay visualization to analyze the co-authorship between institutions, with a minimum article count of 4, and a total of 36 institutions were included in the analysis (Fig. 3A). The thickness of the lines reflected the collaborative strength between institutions. University of Tokyo had the highest total link strength (*n* = 57), followed by Japanese Red Cross Medical Center (*n* = 54) and Musashino Red Cross Hospital (*n* = 54). Johns Hopkins University, Mayo Clinic, Columbia University, and 14 other institutions formed the central cluster. Japanese Red Cross Medical Center, Musashino Red Cross Hospital, University of Tokyo, and eight other institutions constituted another primary cluster with close cooperation among institutions. Analysis of the overlay visualization revealed that Johns Hopkins University and Vanderbilt University were the main institutions studying spinal SSI in the early years (around 2011), while Saitama Rehabilitation Center, Gunma University, and Rothman Institute have become the main forces in recent years (around 2020) (Fig. 3B).

### Journal of publication

All articles were published in 83 academic journals. The top 10 journals accounted for 54.7% of the total publications and 65.8% of the total citations (Table 2). Among them, *Spine* contributed 47 articles (1715 citations), far exceeding *European Spine Journal* (27 articles; 753 citations) and *Spine Journal* (20 articles; 799 citations).

**Table 2:** Journals with more than five publications

Rank	Journal	Publications	Total citations	Mean citations	IF
1	Spine	47	1715	36.49	3.2411
2	European Spine Journal	27	753	27.89	2.7211
3	Spine Journal	20	799	39.95	4.2974
4	World Neurosurgery	16	141	8.81	2.2102
5	Journal of Neurosurgery-Spine	11	595	54.09	3.4669
6	Global Spine Journal	11	148	13.45	2.2301
7	Clinical Spine Surgery	11	52	4.73	1.7228
8	Journal of Pediatric Orthopaedics	9	261	29.00	2.5372
9	Infection Control and Hospital Epidemiology	8	177	22.13	6.5203
10	Surgical Infections	8	110	13.75	1.8532
11	Medicine	7	56	8.00	1.8172
12	Journal of Bone and Joint Surgery-American Volume	6	796	132.67	6.5581
13	Journal of Orthopaedic Science	6	198	33.00	1.8052
14	Journal of Clinical Neuroscience	6	133	22.17	2.1159
15	Journal of Hospital Infection	5	63	12.60	8.9445
16	Clinical Neurology and Neurosurgery	5	55	11.00	1.8850
17	Orthopaedics and Traumatology-Surgery and Research	5	45	9.00	2.4250
18	Journal of Orthopaedic Surgery and Research	5	42	8.40	2.6769

### Keywords analysis and research interest

**Fig. 4:**
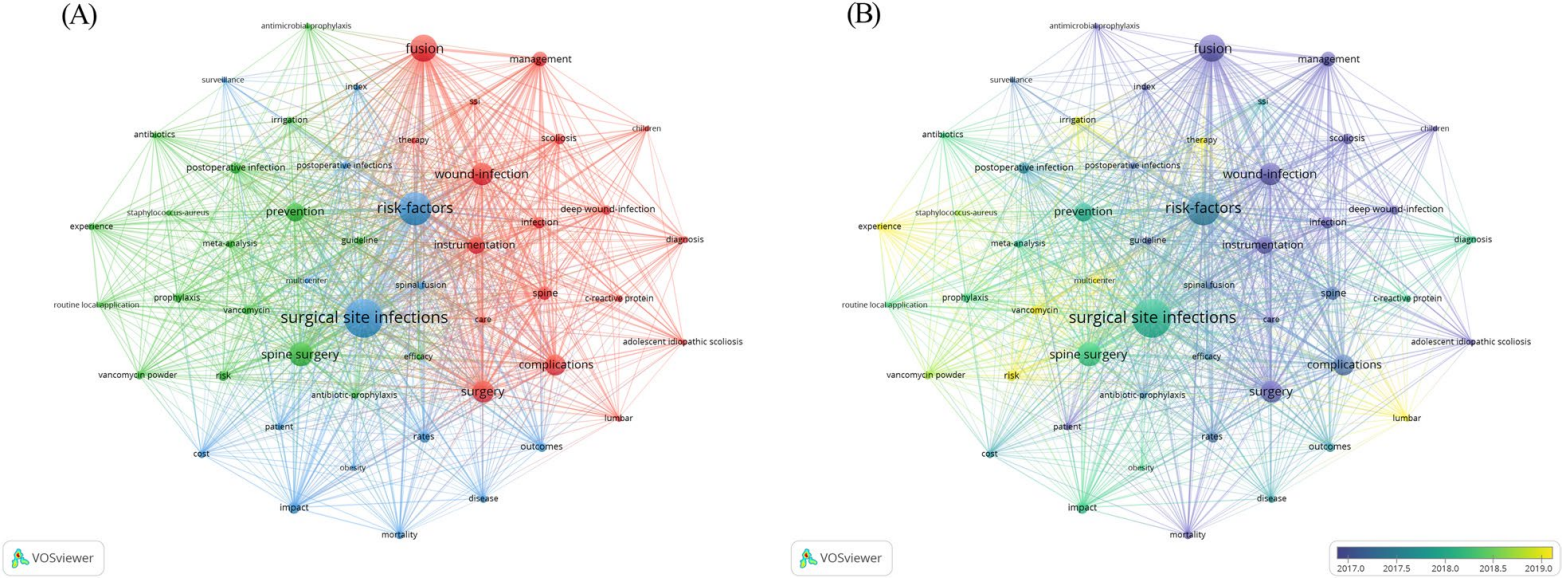
**A** Network visualization map of keywords. The keywords are generally divided into three separate clusters. These clusters represent the sub-subjects of the research **B** Overlay visualization map shows the trend of high-frequency keywords over time

Keywords were analyzed using the network visualization function of VOSviewer, with a minimum word frequency of 10. A total of 50 high-frequency keywords were identified. All keywords were divided into three groups, "prevention," "treatment," and "prognosis," and the same color represented the same research direction. Among the "prevention" group, the keywords with the highest frequency were "spine surgery," "prevention," and "postoperative infection." In the "treatment" group, the most normal keywords were "fusion," "wound-infection," and "surgery." In the "prognostic" group, "surgical site infections," "risk-factors," and "impact" were common (Fig. 4A).

To further explore the evolving trends of keywords over the years, we used overlay visualization to analyze keywords and signify the average year of their appearance through color variation. The shades of purple represent an earlier average year of appearance, around 2017, whereas the shades of yellow indicate a later average year, around 2019. During the initial phase of researching spinal SSI, keywords such as "adolescent idiopathic scoliosis," "care," and "wound-infection" appeared more frequently, while "vancomycin," "risk," and "experience" have emerged as the main focal points of research in recent years (Fig. 4B).

### The 100 most cited articles

**Fig. 5:**
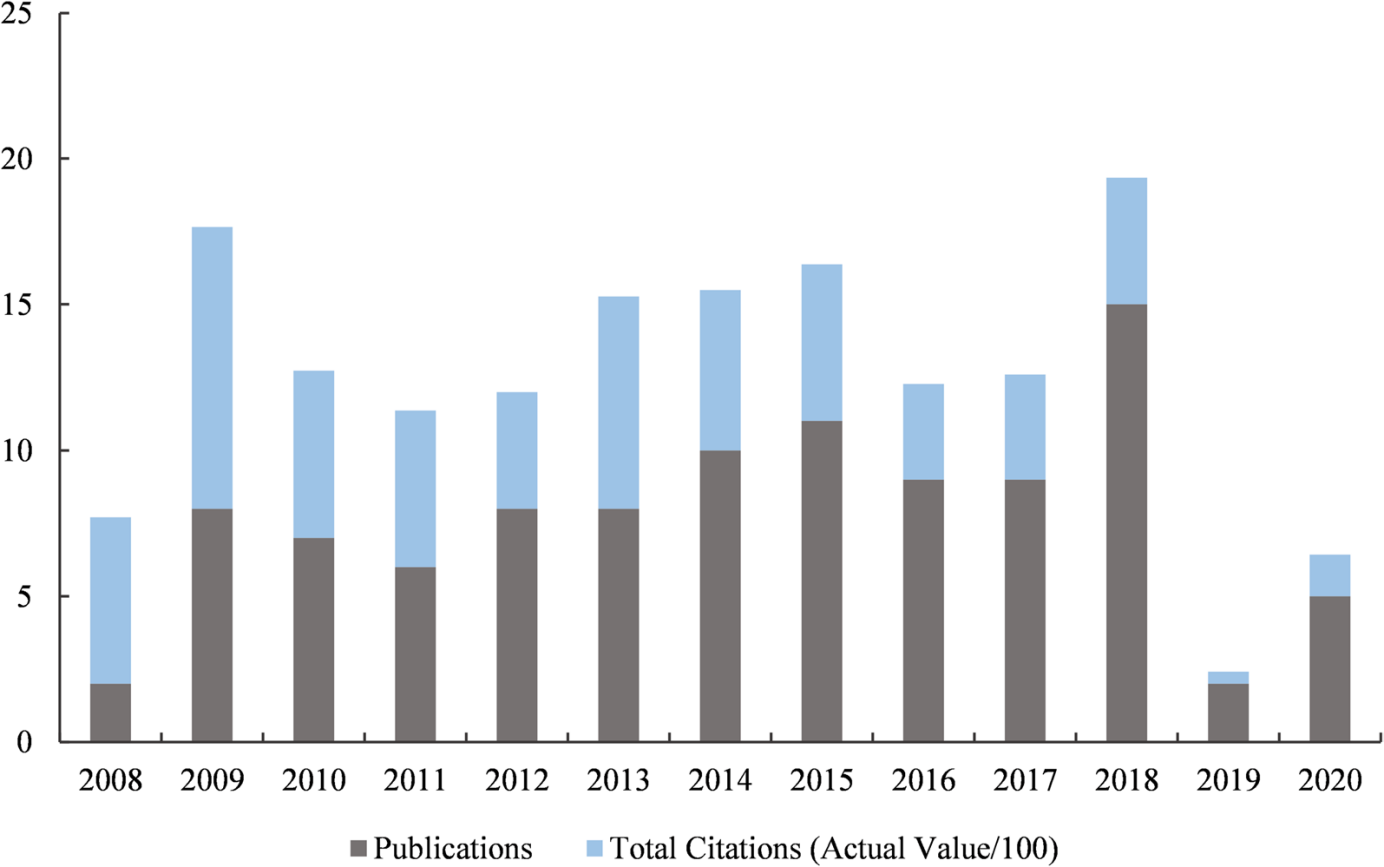
Annual publication volume of the top 100 most cited articles

Analyzing the top 100 cited articles can be used to evaluate the research hotspots and trends in the field. All articles were sorted in descending order of citation frequency, and further analysis was performed on the top 100 articles (Table 3). The top 100 most cited articles were published between 2008 and 2020. Among them, 2018 was the most productive year with 15 articles, followed by 2015 (11 articles) and 2014 (10 articles) (Fig. 5).

**Table 3:** The 100 most cited articles on spinal SSI

Rank	First author	Title	Journal	Citations	Year	Citations per year
1	Olsen, MA	Risk factors for surgical site infection following orthopedic spinal operations	Journal of Bone and Joint Surgery-American Volume	507	2008	33.8
2	ter Gunne, AFP	Incidence, Prevalence, and Analysis of Risk Factors for Surgical Site Infection Following Adult Spinal Surgery	Spine	315	2009	22.5
3	O'Neill, KR	Reduced surgical site infections in patients undergoing posterior spinal stabilization of traumatic injuries using vancomycin powder	Spine Journal	201	2011	16.8
4	O'Toole, JE	Surgical site infection rates after minimally invasive spinal surgery clinical article	Journal of Neurosurgery-Spine	184	2009	13.1
5	Schimmel, JJP	Risk factors for deep surgical site infections after spinal fusion	European Spine Journal	169	2010	13.0
6	Vitale, MG	Building Consensus: Development of a Best Practice Guideline (BPG) for Surgical Site Infection (SSI) Prevention in High-risk Pediatric Spine Surgery	Journal of Pediatric Orthopaedics	135	2013	13.5
7	Rao, SB	Risk Factors for Surgical Site Infections Following Spinal Fusion Procedures: A Case–Control Study	Clinical Infectious Diseases	113	2011	9.4
8	McGirt, MJ	Comparative analysis of perioperative surgical site infection after minimally invasive versus open posterior/transforaminal lumbar interbody fusion: analysis of hospital billing and discharge data from 5170 patients Clinical article	Journal of Neurosurgery-Spine	111	2011	9.3
9	Hedequist, D	Failure of Attempted Implant Retention in Spinal Deformity Delayed Surgical Site Infections	Spine	109	2009	7.8
10	Caroom, C	Intrawound Vancomycin Powder Reduces Surgical Site Infections in Posterior Cervical Fusion	Spine	105	2013	10.5
11	Linam, WM	Risk Factors Associated With Surgical Site Infection After Pediatric Posterior Spinal Fusion Procedure	Infection Control and Hospital Epidemiology	104	2009	7.4
12	Mackenzie, WGS	Surgical Site Infection Following Spinal Instrumentation for Scoliosis	Journal of Bone and Joint Surgery-American Volume	102	2013	10.2
13	Kang, DG	Intrasite vancomycin powder for the prevention of surgical site infection in spine surgery: a systematic literature review	Spine Journal	98	2015	12.3
14	ter Gunne, AFP	The Presentation, Incidence, Etiology, and Treatment of Surgical Site Infections After Spinal Surgery	Spine	98	2010	7.5
15	Abdul-Jabbar, A	Surgical Site Infection in Spinal Surgery Description of Surgical and Patient-Based Risk Factors for Postoperative Infection Using Administrative Claims Data	Spine	97	2012	8.8
16	Anderson, PA	Prevention of Surgical Site Infection in Spine Surgery	Neurosurgery	96	2017	16.0
17	Glotzbecker, MP	What's the Evidence? Systematic Literature Review of Risk Factors and Preventive Strategies for Surgical Site Infection Following Pediatric Spine Surgery	Journal of Pediatric Orthopaedics	96	2013	9.6
18	Abdul-Jabbar, A	Surgical Site Infections in Spine Surgery Identification of Microbiologic and Surgical Characteristics in 239 Cases	Spine	94	2013	9.4
19	Xing, D	A methodological, systematic review of evidence-based independent risk factors for surgical site infections after spinal surgery	European Spine Journal	93	2013	9.3
20	Sebastian, A	Risk factors for surgical site infection after posterior cervical spine surgery: an analysis of 5,441 patients from the ACS NSQIP 2005–2012	Spine Journal	92	2016	13.1
21	Khan, NR	A meta-analysis of spinal surgical site infection and vancomycin powder	Journal of Neurosurgery-Spine	91	2014	10.1
22	Cizik, AM	Using the Spine Surgical Invasiveness Index to Identify Risk of Surgical Site Infection a Multivariate Analysis	Journal of Bone and Joint Surgery-American Volume	90	2012	8.2
23	Watanabe, M	Risk factors for surgical site infection following spine surgery: efficacy of intraoperative saline irrigation Clinical article	Journal of Neurosurgery-Spine	88	2010	6.8
24	Hikata, T	High preoperative hemoglobin A1c is a risk factor for surgical site infection after posterior thoracic and lumbar spinal instrumentation surgery	Journal of Orthopaedic Science	85	2014	9.4
25	ter Gunne, AFP	Incidence of surgical site infection following adult spinal deformity surgery: an analysis of patient risk	European Spine Journal	83	2010	6.4
26	Maragakis, LL	Intraoperative Fraction of Inspired Oxygen Is a Modifiable Risk Factor for Surgical Site Infection after Spinal Surgery	Anesthesiology	81	2009	5.8
27	Chen, S	Diabetes Associated with Increased Surgical Site Infections in Spinal Arthrodesis	Clinical Orthopaedics and Related Research	78	2009	5.6
28	ter Gunne, AFP	A Methodological Systematic Review on Surgical Site Infections Following Spinal Surgery Part 1: Risk Factors	Spine	77	2012	7.0
29	Maruo, K	Outcome and treatment of postoperative spine surgical site infections: predictors of treatment success and failure	Journal of Orthopaedic Science	71	2014	7.9
30	Schwarzkopf, R	Effects of Perioperative Blood Product Use on Surgical Site Infection Following Thoracic and Lumbar Spinal Surgery	Spine	69	2010	5.3
31	Hey, HWD	Is Intraoperative Local Vancomycin Powder the Answer to Surgical Site Infections in Spine Surgery?	Spine	68	2017	11.3
32	Ramo, BA	Surgical Site Infections After Posterior Spinal Fusion for Neuromuscular Scoliosis	Journal of Bone and Joint Surgery-American Volume	68	2014	7.6
33	Abdallah, DY	Body mass index and risk of surgical site infection following spine surgery: a meta-analysis	European Spine Journal	68	2013	6.8
34	Omeis, IA	Postoperative Surgical Site Infections in Patients Undergoing Spinal Tumor Surgery Incidence and Risk Factors	Spine	68	2011	5.7
35	Fei, Q	Risk Factors for Surgical Site Infection After Spinal Surgery: A Meta-Analysis	World Neurosurgery	66	2016	9.4
36	Demura, S	Surgical Site Infection in Spinal Metastasis Risk Factors and Countermeasures	Spine	66	2009	4.7
37	Milstone, AM	Timing of preoperative antibiotic prophylaxis—A modifiable risk factor for deep surgical site infections after pediatric spinal fusion	Pediatric Infectious Disease Journal	64	2008	4.3
38	Meng, F	Risk factors for surgical site infections following spinal surgery	Journal of Clinical Neuroscience	61	2015	7.6
39	Tomov, M	Reducing Surgical Site Infection in Spinal Surgery With Betadine Irrigation and Intrawound Vancomycin Powder	Spine	60	2015	7.5
40	Zhou, JM	Incidence of Surgical Site Infection After Spine Surgery A Systematic Review and Meta-analysis	Spine	59	2020	19.7
41	Radcliff, KE	What is new in the diagnosis and prevention of spine surgical site infections	Spine Journal	59	2015	7.4
42	Yao, R	Surgical Site Infection in Spine Surgery: Who Is at Risk?	Global Spine Journal	55	2018	11.0
43	Nota, SPFT	Incidence of Surgical Site Infection After Spine Surgery: What Is the Impact of the Definition of Infection?	Clinical Orthopaedics and Related Research	53	2015	6.6
44	Lee, MJ	Predicting surgical site infection after spine surgery: a validated model using a prospective surgical registry	Spine Journal	52	2014	5.8
45	Liu, JM	Risk Factors for Surgical Site Infection After Posterior Lumbar Spinal Surgery	Spine	51	2018	10.2
46	Patel, H	Burden of Surgical Site Infections Associated with Select Spine Operations and Involvement of Staphylococcus aureus	Surgical Infections	48	2017	8.0
47	Klemencsics, I	Risk factors for surgical site infection in elective routine degenerative lumbar surgeries	Spine Journal	48	2016	6.9
48	Heller, A	Intrawound Vancomycin Powder Decreases Staphylococcal Surgical Site Infections After Posterior Instrumented Spinal Arthrodesis	Journal of Spinal Disorders and Techniques	48	2015	6.0
49	Kang, BU	Surgical site infection in spinal surgery: detection and management based on serial C-reactive protein measurements Clinical article	Journal of Neurosurgery-Spine	47	2010	3.6
50	Manoso, MW	Medicaid Status Is Associated With Higher Surgical Site Infection Rates After Spine Surgery	Spine	45	2014	5.0
51	Ee, WWG	Does Minimally Invasive Surgery Have a Lower Risk of Surgical Site Infections Compared With Open Spinal Surgery?	Clinical Orthopaedics and Related Research	45	2014	5.0
52	Blumberg, TJ	Predictors of increased cost and length of stay in the treatment of postoperative spine surgical site infection	Spine Journal	41	2018	8.2
53	DiPaola, CP	Postoperative Infection Treatment Score for the Spine (PITSS): construction and validation of a predictive model to define need for single versus multiple irrigation and debridement for spinal surgical site infection	Spine Journal	40	2012	3.6
54	Croft, LD	Risk Factors for Surgical Site Infections After Pediatric Spine Operations	Spine	37	2015	4.6
55	Ogihara, S	Prospective multicenter surveillance and risk factor analysis of deep surgical site infection after posterior thoracic and/or lumbar spinal surgery in adults	Journal of Orthopaedic Science	37	2015	4.6
56	Satake, K	Predisposing factors for surgical site infection of spinal instrumentation surgery for diabetes patients	European Spine Journal	34	2013	3.4
57	Lonjon, G	Early surgical site infections in adult spinal trauma: A prospective, multicentre study of infection rates and risk factors	Orthopaedics and Traumatology-Surgery and Research	34	2012	3.1
58	Atkinson, RA	Management and cost of surgical site infection in patients undergoing surgery for spinal metastasis	Journal of Hospital Infection	32	2017	5.3
59	Thakkar, V	Nasal MRSA colonization: Impact on surgical site infection following spine surgery	Clinical Neurology and Neurosurgery	32	2014	3.6
60	Ando, M	Surgical site infection in spinal surgery: a comparative study between 2-octyl-cyanoacrylate and staples for wound closure	European Spine Journal	32	2014	3.6
61	Pesenti, S	What are the risk factors for surgical site infection after spinal fusion? A meta-analysis	European Spine Journal	31	2018	6.2
62	Tempel, Z	Prealbumin as a Serum Biomarker of Impaired Perioperative Nutritional Status and Risk for Surgical Site Infection after Spine Surgery	Journal of Neurological Surgery Part A-Central European Neurosurgery	31	2015	3.9
63	Horii, C	Does intrawound vancomycin powder reduce surgical site infection after posterior instrumented spinal surgery? A propensity score-matched analysis	Spine Journal	30	2018	6.0
64	Devin, CJ	Intrawound Vancomycin Decreases the Risk of Surgical Site Infection After Posterior Spine Surgery: A Multicenter Analysis	Spine	30	2018	6.0
65	Kong, LD	Smoking and Risk of Surgical Site Infection after Spinal Surgery: A Systematic Review and Meta-Analysis	Surgical Infections	30	2017	5.0
66	Dubory, A	Surgical site infection in spinal injury: incidence and risk factors in a prospective cohort of 518 patients	European Spine Journal	29	2015	3.6
67	Thompson, GH	Does Vancomycin Powder Decrease Surgical Site Infections in Growing Spine Surgery? A Preliminary Study	Journal of Bone and Joint Surgery-American Volume	28	2018	5.6
68	Nunez-Pereira, S	Postoperative urinary tract infection and surgical site infection in instrumented spinal surgery: is there a link?	Clinical Microbiology and Infection	28	2014	3.1
69	Boston, KM	Risk Factors for Spinal Surgical Site Infection, Houston, Texas	Infection Control and Hospital Epidemiology	28	2009	2.0
70	Deng, H	Risk factors for deep surgical site infection following thoracolumbar spinal surgery	Journal of Neurosurgery-Spine	27	2020	9.0
71	Wang, T	Factors predicting surgical site infection after posterior lumbar surgery A multicenter retrospective study	Medicine	26	2017	4.3
72	Nunez-Pereira, S	Individualized antibiotic prophylaxis reduces surgical site infections by gram-negative bacteria in instrumented spinal surgery	European Spine Journal	25	2011	2.1
73	Yin, D	Management of late-onset deep surgical site infection after instrumented spinal surgery	Bmc Surgery	24	2018	4.8
74	Lewkonia, P	Incidence and risk of delayed surgical site infection following instrumented lumbar spine fusion	Journal of Clinical Neuroscience	24	2016	3.4
75	Inanami, H	Role of F-18-Fluoro-D-deoxyglucose PET/CT in Diagnosing Surgical Site Infection After Spine Surgery With Instrumentation	Spine	24	2015	3.0
76	Sebaaly, A	Surgical site infection in spinal metastasis: incidence and risk factors	Spine Journal	23	2018	4.6
77	Tominaga, H	Risk factors for surgical site infection and urinary tract infection after spine surgery	European Spine Journal	23	2016	3.3
78	van Middendorp, JJ	A Methodological Systematic Review on Surgical Site Infections Following Spinal Surgery Part 2: Prophylactic Treatments	Spine	23	2012	2.1
79	Peng, XQ	Risk Factors for Surgical Site Infection After Spinal Surgery: A Systematic Review and Meta-Analysis Based on Twenty-Seven Studies	World Neurosurgery	22	2019	5.5
80	Salvetti, DJ	Low preoperative serum prealbumin levels and the postoperative surgical site infection risk in elective spine surgery: a consecutive series	Journal of Neurosurgery-Spine	22	2018	4.4
81	Yao, RN	Prophylaxis of surgical site infection in adult spine surgery: A systematic review	Journal of Clinical Neuroscience	22	2018	4.4
82	Lai, Q	Risk factors for acute surgical site infections after lumbar surgery: a retrospective study	Journal of Orthopaedic Surgery and Research	21	2017	3.5
83	Tsantes, AG	Association of malnutrition with surgical site infection following spinal surgery: systematic review and meta-analysis	Journal of Hospital Infection	20	2020	6.7
84	Tsubouchi, N	Risk factors for implant removal after spinal surgical site infection	European Spine Journal	20	2018	4.0
85	Haimoto, S	Reduction in surgical site infection with suprafascial intrawound application of vancomycin powder in instrumented posterior spinal fusion: a retrospective case–control study	Journal of Neurosurgery-Spine	20	2018	4.0
86	Van Hal, M	Vancomycin Powder Regimen for Prevention of Surgical Site Infection in Complex Spine Surgeries	Clinical Spine Surgery	20	2017	3.3
87	Ahn, DK	The Difference of Surgical Site Infection According to the Methods of Lumbar Fusion Surgery	Journal of Spinal Disorders and Techniques	20	2012	1.8
88	Tan, T	Prophylactic postoperative measures to minimize surgical site infections in spine surgery: systematic review and evidence summary	Spine Journal	19	2020	6.3
89	Lemans, JVC	Intrawound Treatment for Prevention of Surgical Site Infections in Instrumented Spinal Surgery: A Systematic Comparative Effectiveness Review and Meta-Analysis	Global Spine Journal	19	2019	4.8
90	Spina, NT	Surgical Site Infections in Spine Surgery: Preoperative Prevention Strategies to Minimize Risk	Global Spine Journal	19	2018	3.8
91	Jalai, CM	Surgical site infections following operative management of cervical spondylotic myelopathy: prevalence, predictors of occurrence, and influence on perioperative outcomes	European Spine Journal	19	2016	2.7
92	Floccari, LV	Surgical Site Infections After Pediatric Spine Surgery	Orthopedic Clinics of North America	19	2016	2.7
93	ter Gunne, AFP	Surgical site infection after osteotomy of the adult spine: does type of osteotomy matter?	Spine Journal	19	2010	1.5
94	Agarwal, A	Implant Retention or Removal for Management of Surgical Site Infection After Spinal Surgery	Global Spine Journal	18	2020	6.0
95	Yamada, K	Evidence-based Care Bundles for Preventing Surgical Site Infections in Spinal Instrumentation Surgery	Spine	18	2018	3.6
96	Warner, SJ	Epidemiology of Deep Surgical Site Infections After Pediatric Spinal Fusion Surgery	Spine	18	2017	3.0
97	Haleem, A	Risk Factors for Surgical Site Infections Following Adult Spine Operations	Infection Control and Hospital Epidemiology	18	2016	2.6
98	Ojo, OA	Surgical site infection in posterior spine surgery	Nigerian Journal of Clinical Practice	18	2016	2.6
99	Tofuku, K	The use of antibiotic-impregnated fibrin sealant for the prevention of surgical site infection associated with spinal instrumentation	European Spine Journal	18	2012	1.6
100	Chikawa, T	Retrospective study of deep surgical site infections following spinal surgery and the effectiveness of continuous irrigation	British Journal of Neurosurgery	18	2011	1.5

**Fig. 6:**
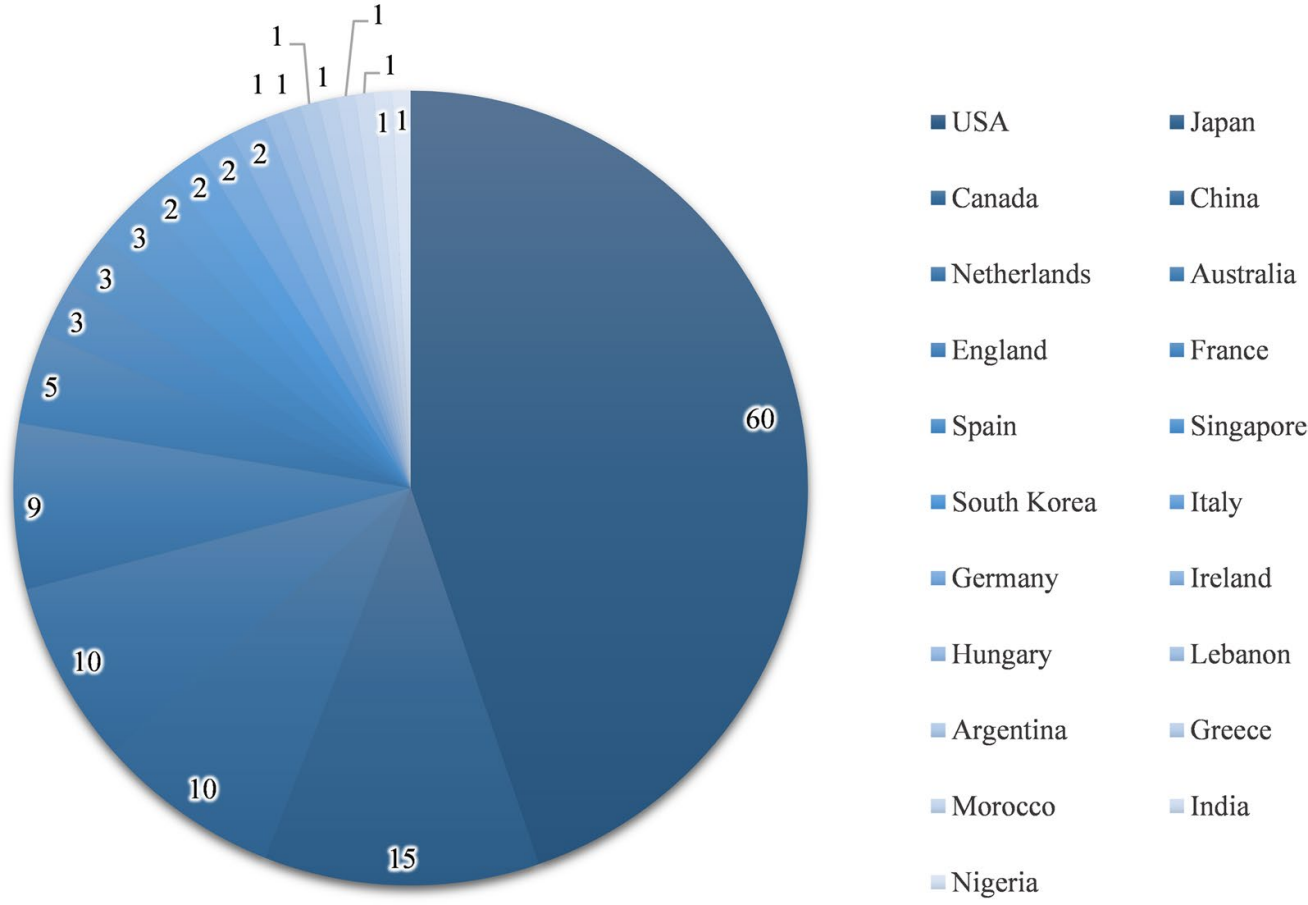
The number of articles published by countries among the top 100 most cited articles

The top 100 cited articles were authored by researchers from 21 different countries. The USA, being the most influential country in the field of spinal SSI, contributed 60 articles (4499 citations), which was significantly more than Japan (15 articles; 584 citations), Canada (10 articles; 482 citations), China (10 articles; 453 citations), and the Netherlands (9 articles; 538 citations) (Fig. 6).

In terms of institution contributions, Johns Hopkins University contributed the most (*n* = 9), followed by Thomas Jefferson University, University of Washington, and University of British Columbia, each with five articles (Table 4).

**Table 4:** The top ten most productive institutions among the top 100 most cited articles

Rank	Institutions	Publications	Total citations	Mean citations	Country
1	Johns Hopkins University	9	828	92.00	USA
2	Thomas Jefferson University	5	330	66.00	USA
3	University of Washington	5	255	51.00	USA
4	University of British Columbia	5	160	32.00	Canada
5	Vanderbilt University	4	526	131.50	USA
6	Harvard University	4	393	98.25	USA
7	University of California San Francisco	4	289	72.25	USA
8	University of Tokyo	4	109	27.25	Japan
9	University of Pittsburgh	4	100	25.00	USA
10	Washington University (WUSTL)	3	740	246.67	USA

Regarding journal contributions, the 100 most cited articles were published in 30 different journals. The most productive journal was *Spine*, with a total number of 21 articles (1531 citations), followed by *European Spine Journal* (13 articles; 644 citations), *Spine Journal* (12 articles; 722 citations), *Journal of Neurosurgery-Spine* (eight articles; 590 citations), and *Journal of Bone and Joint Surgery-American Volume* (five articles; 795 citations) (Table 5).

**Table 5:** The number of articles published in each journal among the top 100 most cited articles

Rank	Journal	Publications	Total citations	Mean citations	IF
1	Spine	21	1531	72.90	3.2411
2	European Spine Journal	13	644	49.54	2.7211
3	Spine Journal	12	722	60.17	4.2974
4	Journal of Neurosurgery-Spine	8	590	73.75	3.4669
5	Journal of Bone And Joint Surgery-American Volume	5	795	159.00	6.5581
6	Global Spine Journal	4	111	27.75	2.2301
7	Journal of Clinical Neuroscience	3	107	35.67	2.1159
8	Clinical Orthopaedics and Related Research	3	176	58.67	4.7552
9	Journal of Orthopaedic Science	3	193	64.33	1.8052
10	Infection Control and Hospital Epidemiology	3	150	50.00	6.5203

In terms of authors, the most productive authors were Cohen, David B. and ter Gunne, Albert F. Pull, both of whom published six articles, followed by McGirt, Matthew J. and Vaccaro, Alexander R. (each four articles) (Table 6).

**Table 6:** The most productive authors among the top 100 most cited articles

Author	Publications	First author	Corresponding author	Total citations	Mean citations
Cohen, David B	6	1	0	619	103.17
ter Gunne, Albert F. Pull	6	1	5	615	102.50
McGirt, Matthew J	4	0	1	410	102.50
Vaccaro, Alexander R	4	0	0	217	54.25

**Fig. 7:**
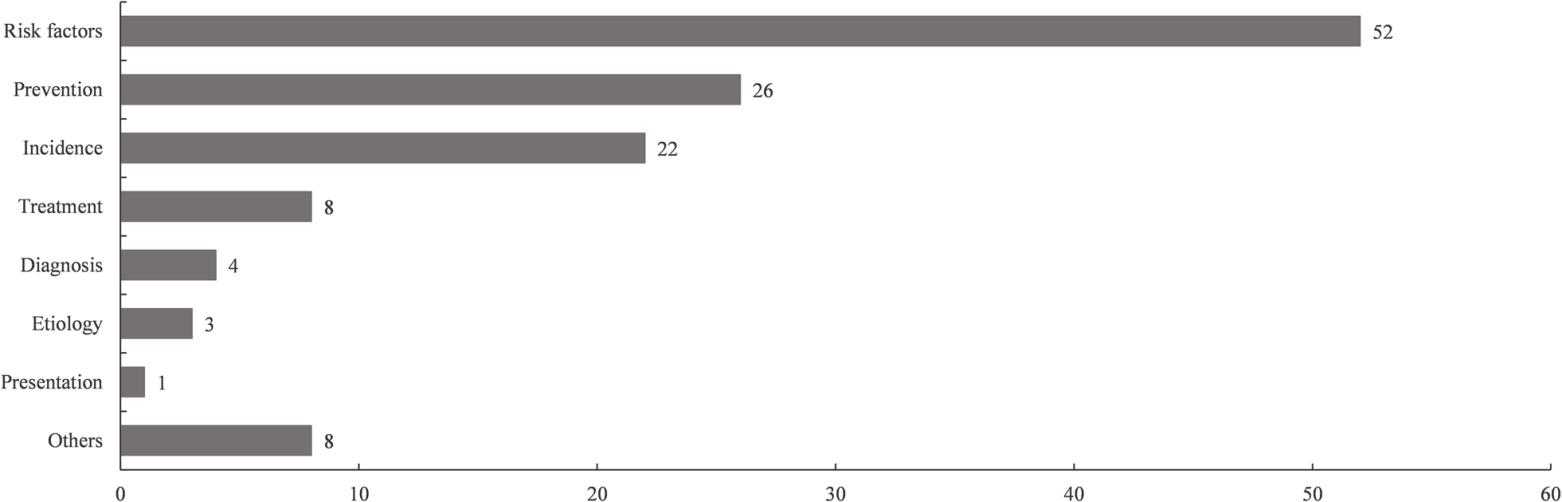
Research topics of the top 100 most cited articles

The most common research topic was Risk Factors for spinal SSI (*n* = 52), followed by Prevention (*n* = 26) and Incidence (*n* = 22) (Fig. 7). Notably, eight of the 100 articles were related to spinal SSI in pediatrics.

## Discussion

As one of the most common complications after spinal surgery, SSI can prolong the disease course, increase medical costs, and affect the prognosis, potentially even leading to neurological dysfunction, paraplegia, and death. The reported incidence of spinal SSI ranges from 0.7 to 16.1%. Early debridement and intravenous antibiotics are effective treatments for spinal SSI. However, the presence of implants can impede the efficacy of antibiotics on bacteria by allowing the formation of a bacterial biofilm [8]. Meanwhile, due to the intricate structure of the biofilm, antibiotics can only eradicate the planktonic and outer-layer bacteria. The eradication of the inner-layer bacteria is currently a challenge, leading to a recurrence of infection [9]. The management of chronic SSI typically involves multiple debridements and, in severe cases, implant removal. Therefore, the primary focus of managing spinal SSI is on implementing multiple measures for comprehensive prevention, aiming to reduce the incidence of SSI and improve patient prognosis. In recent years, publications on spinal SSI have increased steadily. However, an analysis of the current research status and trends in this field remains lacking. As the first article to analyze SSI in spine surgery with bibliometric analysis, the purpose of our study is to provide a practical guide for clinicians to familiarize this field and improve their vigilance toward SSI.

### Publication trends on spinal SSI

Over the past 15 years, publications related to spinal SSI have increased steadily. In particular, the annual publication rate has been consistently above 35 articles in the past three years, indicating a growing interest in research related to spinal SSI. This may be related to the increased morbidity caused by comorbid factors such as diabetes, antibiotic abuse, immune suppression, and spinal instrumentation, as well as the improvement of diagnostic sensitivity [10]. As a global research center for spinal SSI, the USA has contributed 37% of the total article count and 56% of the total citation count, demonstrating its dominant position in this field. Among all institutions, Johns Hopkins University has contributed the most, with a total of 14 articles and 835 citations. However, the institution with the highest average citation count was Washington University, highlighting its significance in spinal SSI research. The top five journals in terms of publication volume were *Spine*, *European Spine Journal*, *Spine Journal*, *World Neurosurgery*, and *Journal of Neurosurgery-Spine*. However, among journals with more than five publications, the one with the highest average citation frequency was *Journal of Bone and Joint Surgery-American Volume* (132.7 times). Clinicians and scholars interested in spinal SSI should pay more attention to the abovementioned journals. It is noteworthy that the institutions and journals mentioned above are all affiliated with the USA, which explains why it occupies a dominant position in this field.

### Research focuses

Keywords represent the core of the article, and the evolution in keywords over time can reflect the research trends in that field. The network visualization analysis of the keywords demonstrated that "risk-factors," "prevention," and "fusion" were the centers of the keyword clusters. As a common cause of C (HAI) and death, SSI accounts for roughly 20% of all HAIs [11]. The incidence of SSI in spinal surgery is approximately 2%, and it has shown an upward trend in recent years due to the extensive use of implants and the increased complexity of surgical procedures [12]. Smith et al. [13] evaluated the risk factors relevant to spinal SSI and found that the incidence of wound infection was significantly higher in patients who underwent spinal fusion or instrumentation, which may be attributed to the increased risk and complexity of the procedures. Spinal SSI can lead to instrumentation failure, neurological dysfunction, paraplegia, and even death, causing catastrophic consequences for patients. Due to the heterogeneity of patients and the diversity of treatments, there is currently a lack of universally applicable management guidelines for spinal infections [14]. Therefore, the focus of SSI management is on multifactorial comprehensive management with an emphasis on prevention, including preoperative risk stratification and intraoperative measures [15].

We utilized the overlay visualization function to analyze the trends in research hotspots over time. "Fusion" was the center of early keywords and was closely linked with other keywords such as "Instrumentation" and "Scoliosis" during the same period, indicating that the etiology of spinal SSI was the focus of early research. Compared to other orthopedic cleaning surgeries, spinal instrumentation surgery has a higher infection rate [16]. Based on a study of 108,419 cases by Justin S. Smith et al., patients with spinal scoliosis had a higher infection rate (3.7%) than those with degenerative spinal diseases (1.4%), spondylolisthesis (2.1%), and fractures (2.0%). In addition, patients who underwent spinal fusion had a 33% higher infection rate than those who did not undergo fusion (2.4% vs. 1.8%, *P* < 0.001), and patients with implants had a 28% higher infection rate than those without implants (2.3% vs. 1.8%, *P* < 0.001), which is likely related to the higher complexity and risk of instrumentation surgery [13]. Notably, pediatric spinal SSI has attracted the attention of scholars in the early stage of research (Avg. pub. Year: 2017.00). As mentioned above, eight of the top 100 cited articles were related to pediatrics, indicating that pediatric SSI may have its specificity. Cahill et al. [17] found that, unlike adults, the infection risk after spinal surgery in pediatric patients was inversely proportional to age, which meant younger patients have a higher infection rate. Furthermore, diagnosis and treatment in pediatrics are more challenging due to factors such as poor expression ability, low positive blood cultures, lack of specific early symptoms, and imaging features [18]. Therefore, laboratory and imaging examinations are the mainstays of early diagnosis in pediatrics spinal SSI. C-reactive protein (CRP), erythrocyte sedimentation rate (ESR), and absolute neutrophil count (ANC) are commonly used laboratory indicators. However, confounding factors after spinal surgery often affect their sensitivity and specificity. In recent years, studies have suggested that markers such as c (SAA), procalcitonin (PCT), interleukin-6 (IL-6), and leukocyte esterase can serve as crucial auxiliary diagnosis modalities for spinal SSI, with better predictive effect than CRP and leukocyte levels [15, 19].

In recent years, the high-frequency keywords have shifted to such as "risk," "vancomycin," and "experience," indicating a shift in the research focus of spinal SSI from etiology to prevention. According to the estimation by the Society for Healthcare Epidemiology of America (SHEA), up to 60% of SSIs can be prevented by following evidence-based guidelines [20]. The prevention of spinal SSI focuses on the risk factors, and numerous scholars have conducted detailed and comprehensive research on related factors. Currently, well-established factors that are significantly associated with SSI include diabetes, obesity, smoking, previous history of SSI, increased intraoperative blood loss, and prolonged operation time, among others [5, 21]. Preoperative, intraoperative, and postoperative prevention measures are another crucial part of reducing the incidence of SSI. Typical prevention measures include intrawound vancomycin powder, prophylactic antibiotics, closed-suction drainage, povidone–iodine irrigation, and incision closure with 2-octyl-cyanoacrylate [22]. Among them, intrawound vancomycin powder is the most extensively studied prevention measure. As previously mentioned, among the top 100 cited articles, 26 focused on prevention, including 12 related to vancomycin (Fig. 7). Since its first application as a local preventive measure for spinal SSI in 2011, vancomycin has been demonstrated to significantly reduce the incidence of SSI with a favorable safety profile [23]. However, the optimal dose and application site of vancomycin powder are still controversial and need further experimental research [24]. Notably, vancomycin powder has also been confirmed to be safe in children with spinal injuries [25].

### The most influence articles

The frequency of citations represents the degree of recognition of an article in the field, which can roughly reflect the quality and influence of the article.

The most cited article in the field of spinal SSI is "Risk factors for surgical site infection following orthopaedic spinal operations," published by Olsen et al. [12] in *Journal of Bone and Joint Surgery-American Volume* in 2008, with a total of 507 citations. This retrospective case–control study analyzed 2316 postoperative spinal patients, among whom 46 were diagnosed with SSI, and 227 patients without SSI were selected as the control group. A comprehensive and in-depth analysis of the independent risk factors for SSI after spinal surgery was performed using univariate and multivariate logistic regression analyses. The overall incidence of SSI was 2.0%, with a superficial SSI rate of 0.8% (18 cases), a deep SSI rate of 0.9% (20 cases), and an organ space SSI rate of 0.3% (8 cases). The study identified diabetes, poor timing of prophylactic antibiotics, hyperglycemia, obesity, and the involvement of two or more residents as independent risk factors for SSI in laminectomy, discectomy, and spinal fusion. In contrast, cervical spine surgery was independently associated with a significantly lower risk of SSI. Among all independent risk factors, diabetes had the strongest correlation with spinal SSI, followed by poor timing of prophylactic antibiotics and hyperglycemia.

The second most cited article is "Incidence, Prevalence, and Analysis of Risk Factors for Surgical Site Infection Following Adult Spinal Surgery" by ter Gunne, Albert et al. [6], published in *Spine* in 2009, with a total of 315 citations. The purpose of this study was to compare infected patients with uninfected patients through a retrospective cohort study, to calculate the incidence of SSI and identify the risk factors for postoperative wound infection after spinal surgery. A total of 3174 patients were enrolled in the study. There were 132 cases of SSI (4.2%), including 70 cases of superficial SSI (2.2%) and 84 cases of deep SSI (2.6%). Multivariate logistic regression analysis revealed that estimated blood loss (EBL) exceeding 1L, previous history of SSI, and diabetes were independent risk factors for spinal SSI. Analysis of superficial SSI showed that obesity, hypertension, multilevel fusion, surgical approach, and operative time longer than 2 h were associated with an increased risk of superficial SSI. However, only obesity significantly increased the risk of superficial infection, while the anterior surgical approach significantly reduced the risk of SSI. In deep infection, diabetes, obesity, previous history of SSI, surgery for spinal deformity, multilevel fusion, surgical approach, spinal uninstrumented fusion, osteotomies, and operative time longer than 2 h were associated with a higher infection rate. In comparison, discectomy alone and the anterior surgical approach had a lower infection rate. Among all risk factors, diabetes, obesity, previous history of SSI, and operative time longer than 2 h were independent risk factors for deep SSI.

"Reduced surgical site infections in patients undergoing posterior spinal stabilization of traumatic injuries using vancomycin powder," published in *The Spine Journal* by Devin, Clinton J. et al. in 2011, ranked third with 201 citations [26]. In this retrospective cohort study, 110 patients who underwent posterior spinal stabilization due to trauma were analyzed to evaluate the clinical efficacy of topical intrawound vancomycin powder to prevent SSI. All patients accepted standard intravenous antibiotic prophylaxis, on which cases were divided into a treatment group (with topical vancomycin; *n* = 56) and a control group (without topical vancomycin; *n* = 54). There were no statistical differences in age, BMI, surgical levels, or other patient parameters between the two groups. The study found that none of the patients with topical vancomycin prophylaxis suffered SSI and had no adverse reactions, while the control group had seven SSIs (13%), including two superficial and five deep. The study concluded that topical vancomycin powder could significantly reduce the incidence of SSI in patients with spinal trauma, which was consistent with previous studies [27]. The limitation of this study was the potential mismatch between the groups. The operation time in the control group was significantly longer than that in the treatment group (*P* = 0.01), and it was unclear whether it affected the incidence of SSI in the control group.

Among the top 100 cited articles, the latest article is "Incidence of Surgical Site Infection After Spine Surgery A Systematic Review and Meta-analysis" by Zhou et al., [5] published in *Spine* in 2020. This study conducted a meta-analysis of 27 studies related to spinal SSI, with subgroup analyses for SSI type, age, BMI, diagnosis, surgical site, approach, procedure, minimally invasive or not, operative time, blood loss, topical vancomycin powder, and bacterial culture results. The total incidence of SSI was 3.1% (603 of 22,475 cases), with a superficial SSI of 1.4% and a deep SSI of 1.7%. Among all primary diseases, patients with neuromuscular scoliosis had the highest incidence of SSI (13.0%), while patients with idiopathic scoliosis had the lowest infection rate (2.6%). Subgroup analysis indicated that the incidence of SSI after thoracic surgery (3.7%) was slightly higher than that of cervical (3.4%) and lumbar (2.7%) surgery. Besides, posterior surgery, instrumented surgery, traditional open spinal surgery, age over 60 years, prolonged operation time (≥ 3 h), and increased intraoperative blood loss (> 500 ml) all increase the risk of SSI. Topical vancomycin powder significantly reduced the risk and incidence of SSI (1.9% vs. 4.8%). Nevertheless, further research is needed to confirm the specific efficacy and potential side effects of topical vancomycin. Microbiological cultures suggested that the detection rate of *Staphylococcus* was the highest (50.2%), with *Staphylococcus aureus* and *Staphylococcus epidermidis* being the most common pathogenic bacteria.

### Limitations

This article has several limitations. Firstly, we retrieved from the database in English, and influential articles in other databases or non-English languages may be omitted. Secondly, like other bibliometric studies, our study will inevitably have a "cumulative effect" and may omit recent influential articles. Finally, citation frequency is not the only criterion to evaluate the quality and influence of articles. In future studies, other factors should be considered comprehensively.

## Conclusion

The bibliometric analysis of spinal SSI indicated that the number of publications had shown a steady growth trend over the past 15 years, especially in the past three years, with an annual publication value of more than 35 articles. As the global research center for spinal SSI, the USA contributed the most and had absolute authority in this field. Johns Hopkins University, Mayo Clinic, and University of Tokyo were the institutions with the highest number of publications, while *Spine*, *European Spine Journal*, and *Spine Journal* were the top three contributed journals. The research trend has transitioned from etiology to prophylaxis. Prevention has been a recent hotspot in spinal SSI research, including preoperative risk factors and perioperative preventive measures. Besides, we have listed the 100 top-cited articles to provide a reference for clinicians interested in this field.

## Data Availability

The data involved in this study are available from the Web of Science, www.webofknowledge.com.

## Abbreviations

ANC: Absolute neutrophil count
CRP: C-reactive protein
EBL: Estimated blood loss
ESR: Erythrocyte sedimentation rate
HAI: Estimated blood loss
IL-6: Interleukin-6
PCT: Procalcitonin
SAA: Absolute neutrophil count
SHEA: Healthcare Epidemiology of America
SSI: Surgical site infection
